# The Structural Basis for Activation and Inhibition of ZAP-70 Kinase Domain

**DOI:** 10.1371/journal.pcbi.1004560

**Published:** 2015-10-16

**Authors:** Roland G. Huber, Hao Fan, Peter J. Bond

**Affiliations:** 1 Bioinformatics Institute (BII), Agency for Science, Technology and Research (A*STAR), Singapore; 2 Department of Biological Sciences, National University of Singapore, Singapore; University of North Carolina at Chapel Hill, UNITED STATES

## Abstract

ZAP–70 (Zeta-chain-associated protein kinase 70) is a tyrosine kinase that interacts directly with the activated T-cell receptor to transduce downstream signals, and is hence a major player in the regulation of the adaptive immune response. Dysfunction of ZAP–70 causes selective T cell deficiency that in turn results in persistent infections. ZAP–70 is activated by a variety of signals including phosphorylation of the kinase domain (KD), and binding of its regulatory tandem Src homology 2 (SH2) domains to the T cell receptor. The present study investigates molecular mechanisms of activation and inhibition of ZAP–70 via atomically detailed molecular dynamics simulation approaches. We report microsecond timescale simulations of five distinct states of the ZAP–70 KD, comprising apo, inhibited and three phosphorylated variants. Extensive analysis of local flexibility and correlated motions reveal crucial transitions between the states, thus elucidating crucial steps in the activation mechanism of the ZAP–70 KD. Furthermore, we rationalize previously observed staurosporine-bound crystal structures, suggesting that whilst the KD superficially resembles an “active-like” conformation, the inhibitor modulates the underlying protein dynamics and restricts it in a compact, rigid state inaccessible to ligands or cofactors. Finally, our analysis reveals a novel, potentially druggable pocket in close proximity to the activation loop of the kinase, and we subsequently use its structure in fragment-based virtual screening to develop a pharmacophore model. The pocket is distinct from classical type I or type II kinase pockets, and its discovery offers promise in future design of specific kinase inhibitors, whilst mutations in residues associated with this pocket are implicated in immunodeficiency in humans.

## Author Summary

ZAP–70 is a key protein kinase in the adaptive immune system. It is essential for development and function of T cells and natural killer cells, and associated mutations can lead to conditions such as severe combined immunodeficiency (SCID). Here, simulations of the ZAP–70 kinase domain are used to study its dynamics in response to different mechanistic signals. We identify crucial motions over microsecond timescales, which help to rationalize in atomic detail previous structural and experimental data regarding its biological regulation. We subsequently propose a scheme for the phosphorylation-dependent activation cascade of ZAP–70, and for its ligand-dependent inhibition. Finally, we characterize a novel cryptic pocket adjacent to the active site and activation loop, which is distinct from classical type I or type II kinase sites. The pocket is in close proximity to several residues whose mutations cause SCID in humans, and its identification offers promise in future drug design efforts.

## Introduction

ZAP–70 is part of the Syk family of protein kinases, and a key player in the adaptive immune system. [1] It is expressed in T cells and natural killer cells [2] and is essential for their development and function. Inactivating mutations of ZAP–70 cause selective T cell deficiency in humans, which in turn leads to conditions such as severe combined immunodeficiency (SCID) [3] and persistent infections [4]. Stimulation of the T cell antigen receptor leads to phosphorylation of tyrosines on intracellular ITAM sequences, favoring recruitment of ZAP–70 via its tandem SH2 domains, and biasing ZAP–70 towards non-auto-inhibited states [5]. Subsequent phosphorylation and auto-phosphorylation events lead to up-regulation of ZAP–70 kinase. In particular, phosphorylation of ZAP–70 at specific tyrosine sites in its KD [6] is likely responsible for its full activation [7]. Mutations of residues Y492 and Y493 in the activation loop of the ZAP–70 KD reveal distinct results for the adjacent phosphorylation sites. The Y492F mutation increases ZAP–70 activity, suggesting that phosphorylation of Y492 is not necessary for the biological function of ZAP–70. However, the Y493F mutation impairs kinase activation. [8] Downstream targets phosphorylated by ZAP–70 comprise SH2 domain containing leukocyte protein (SLP–76) [9] and an integral membrane protein, linker for activation of T cells (LAT) [10]. Both protein targets are crucial for T cell receptor function, and are responsible for downstream signaling and gene transcription.

**Fig 1:**
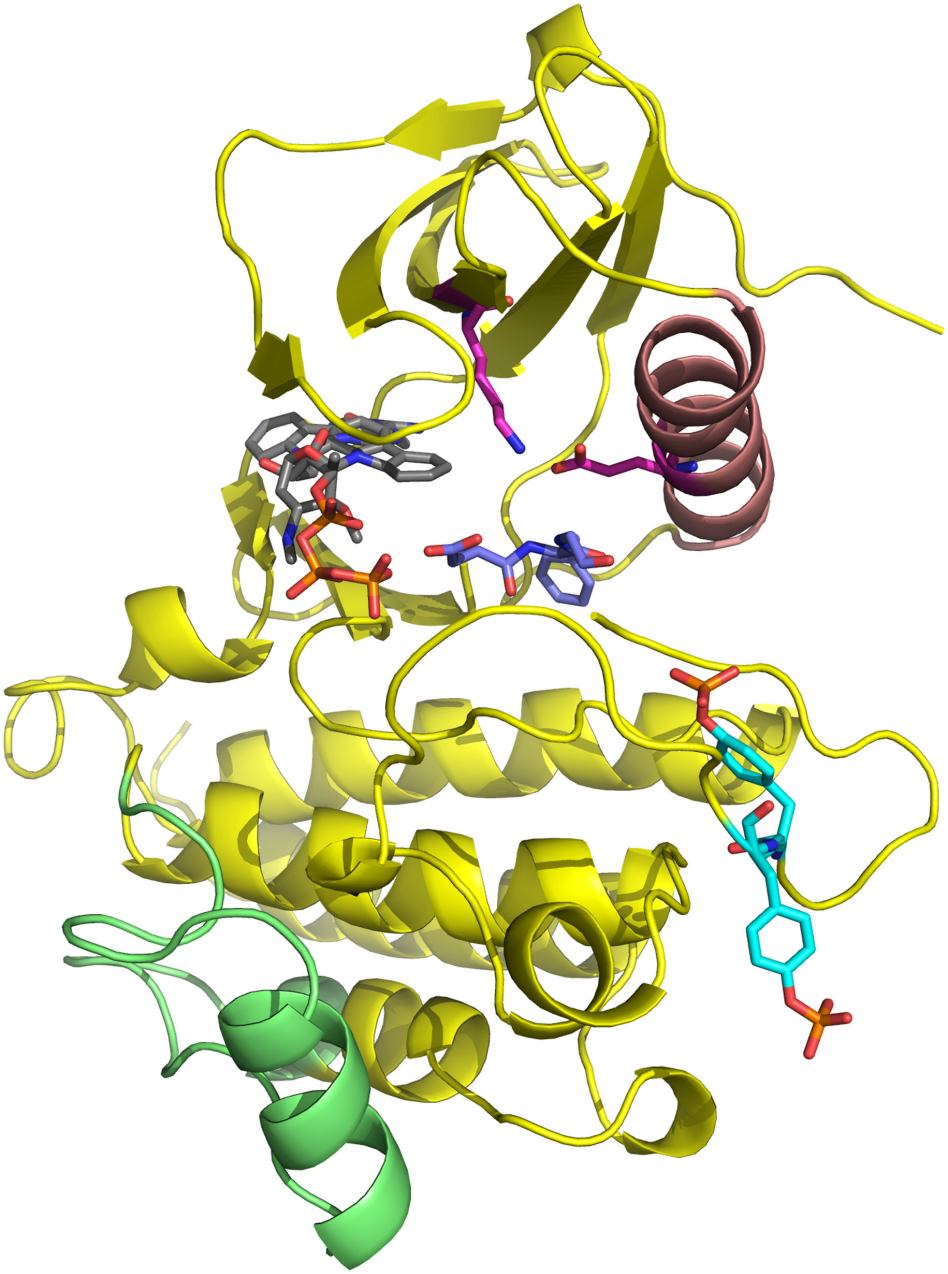
Overview of ZAP–70 kinase domain (PDB 1U59 [11]): Ligands staurosporine or ATP are located in the hinge region between the C-lobe (top) and N-lobe (bottom) of the protein (cartoons format) and are depicted in gray/CPK wireframe format. The αC helix, depicted in red, contains the salt bridge K369-E386 indicated in magenta/CPK wireframe. Phosphorylation sites Y492 and Y493 are indicated in cyan/CPK wireframe. The DFG motif, D479, F480 and G481, is indicated in blue/CPK wireframe. The activation loop comprises all residues from the DFG motif to seven residues beyond the phosphorylation sites. The N-lobal region of residues 537–569 (green) exhibits significant changes in flexibility depending upon phosphorylation state.

Available three-dimensional structures for the ZAP–70 KD include the isolated domain in complex with staurosporine (PDB 1U59 [11]), a well-characterized ATP-competitive inhibitor of kinases [11–12]. Moreover, structures of the full-length complex of ZAP–70 are available, with the KD bound to ANP, but auto-inhibited by its tandem of SH2 domains. The first such structure (PDB 2OZO [13]) included mutations which masked an inhibitory interface between regulatory domain and KD resolved in a subsequent, otherwise similar wild-type structure (PDB 4K2R [14]). Fig 1 illustrates the architecture of the isolated, inhibited KD and highlights significant functional regions. The domain exhibits the distinct bilobal architecture common to other protein kinases, with the activation loop containing Y492 and Y493 located between the two lobes. Staurosporine occupies the ATP binding pocket, which is located at the linkage region between lobes. Despite being bound to inhibitor, Jin et al. reported that the KD is in an active-like state, due to the conformation of the activation loop resembling the geometry of active states observed in the Syk kinase family. However, they noted that the activation loop forms a crystal contact in their structure. Hence, it is unclear whether the loop in the isolated staurosporine complex would likewise adopt an active conformation. Similar conformations of the non-phosphorylated activation loop have been observed for Chk1-staurosporine complexes, although these also involved crystal contacts (PDB 1NVR [15]). The salt bridge formed between K369 and E386 (residue numbers for ZAP–70), located in the αC helix comprising residues D379 to Q392, is a conserved motif of active kinase conformations [16] that is normally broken in inactive kinase states, but was formed in the staurosporine ZAP–70 complex. Deindl et al. observed striking similarities between auto-inhibited ZAP–70 and inhibited Hck [17] and c-Src kinase [18] structures. In comparison to the staurosporine complex, the auto-inhibited, ANP-bound crystal structures of ZAP–70 revealed that the αC helix is displaced outwards leading to a loss of this key salt bridge.

Available crystal structures presently offer an inconclusive picture of the relation of the ZAP–70 KD conformation to the various intermediate mechanistic states. As observed by Jin et al., the staurosporine-bound ZAP–70 KD surprisingly appears to adopt an active-like state when compared to other members of the Syk kinase family. Moreover, experimental data from Chan et al. suggests that Y493 phosphorylation is important for catalytic activity of ZAP–70 whereas the neighboring Y492 is not. Motivated by these observations, we have investigated the activation and inhibition mechanisms of ZAP–70 by using molecular simulations of its KD in a number of mechanistic states, over the microsecond timescales necessary to observe key conformational transitions. We discern structural and dynamic properties that yield a molecular basis for previous biophysical and structural experiments, allowing us to rationalize the inhibitor-bound, superficially “active-like” conformation of ZAP–70, and to propose a scheme for the activation cascade by tracing the phosphorylation-dependent effects through the ZAP–70 KD. Finally, we identify a novel, spontaneously formed cryptic pocket restricted to the non-phosphorylated inactive state of the KD, and use this in virtual fragment-based screening to build a pharmacophore model. The pocket is distinct from classical type I or type II kinase pockets, and hence offers promise in future design of specific kinase inhibitors.

## Results

We calculated a series of 1.5 μs simulation trajectories for each of five states of ZAP–70 KD, comprising either the non-phosphorylated staurosporine-bound complex (STA), non-phosphorylated ATP-bound complex Y^0^Y^0^, the ATP-bound Y492 (Y^P^Y^0^) and Y493 (Y^0^Y^P^) phosphorylated variants, and the Y492+Y493 di-phosphorylated variant Y^P^Y^P^. We analyzed these trajectories with regard to stability, intrinsic flexibility, and presence of characteristic interactions and structural features for both the DFG motif and the αC helix. Moreover, normal mode analysis was performed for the Cα atoms of all trajectories in order to identify state-specific global motions. These motions were then correlated with structural and dynamic information resulting from analysis of the individual states, yielding a coherent picture for the mechanistic steps involved in catalytic (auto)inhibition and activation.

### Staurosporine Complex STA

**Fig 2:**
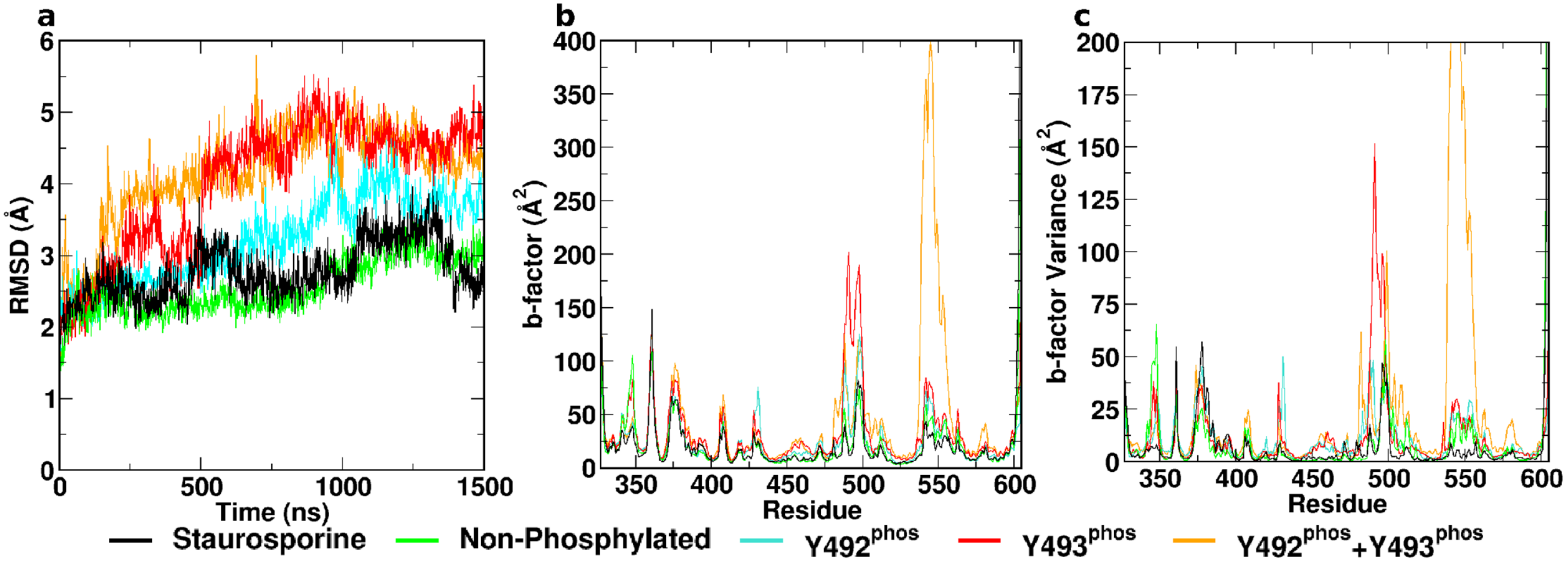
Conformational dynamics of ZAP–70 kinase domain. (a) Cα RMSD, (b) B-factors over final 500 ns, and (c) B-factor variance over 10 sequential independent trajectory segments of all investigated systems. All phosphorylated states exhibit elevated flexibility compared to the staurosporine-inhibited and the ATP-bound non-phosphorylated complexes. Regions of interest comprise the N-lobal residues 500–519 and the “flap-like” residues 537–569.

**Fig 3:**
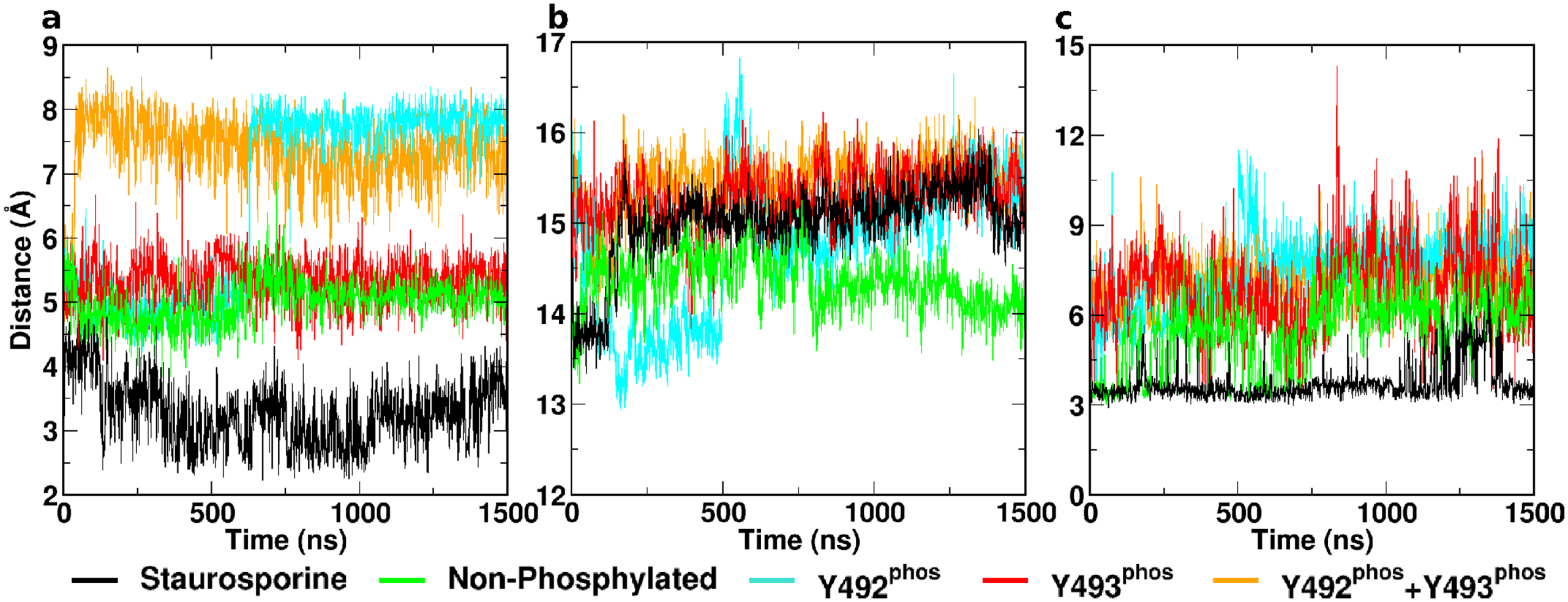
Structural motifs of ZAP–70 kinase domain as observed in the different states. Time-dependent distances are shown for: (a) the DFG motif D479 carboxylate carbon to the G481 backbone amide hydrogen; (b) the αC helix to the C-lobe center of mass; and (c) the salt bridge K369-E386.

The staurosporine-bound complex remained stable throughout the course of the simulation. This is illustrated by the constant Cα-RMSD (Fig 2a), which rapidly plateaued at ~2–3 Å, as well as the low B-factors (Fig 2b) across the entire domain and associated variance throughout the trajectory (Fig 2c). Residual flexibility was observed within the DFG motif, the activation loop downstream of the unmodified phosphorylation sites Y492 and Y493 and the solvent-exposed regions of the αC helix. The DFG motif was observed to form a stable, closed loop structure through a hydrogen bond from the side chain carboxylate-oxygen on D479 through the backbone amide hydrogen on G481 (Fig 3a). This closed loop structure is also present in the staurosporine X-ray structure, 1U59. The distance of the αC helix from the center of mass of the C-lobe initially increased from a value of 14 Å (in the crystallographic state) to 15 Å within ~150 ns and remained stable at this level for the remainder of the trajectory (Fig 3b). Moreover, the phenyl ring of F480 of the DFG motif forms a close contact with the backbone amide of M390 within the αC helix (S1 Fig). The salt bridge K369-E386 was nevertheless present during the entire course of the simulation (Fig 3c). It is present in the staurosporine complex structure 1U59 but absent in the ANP complexes 2OZO and 4K2R.

**Fig 4:**
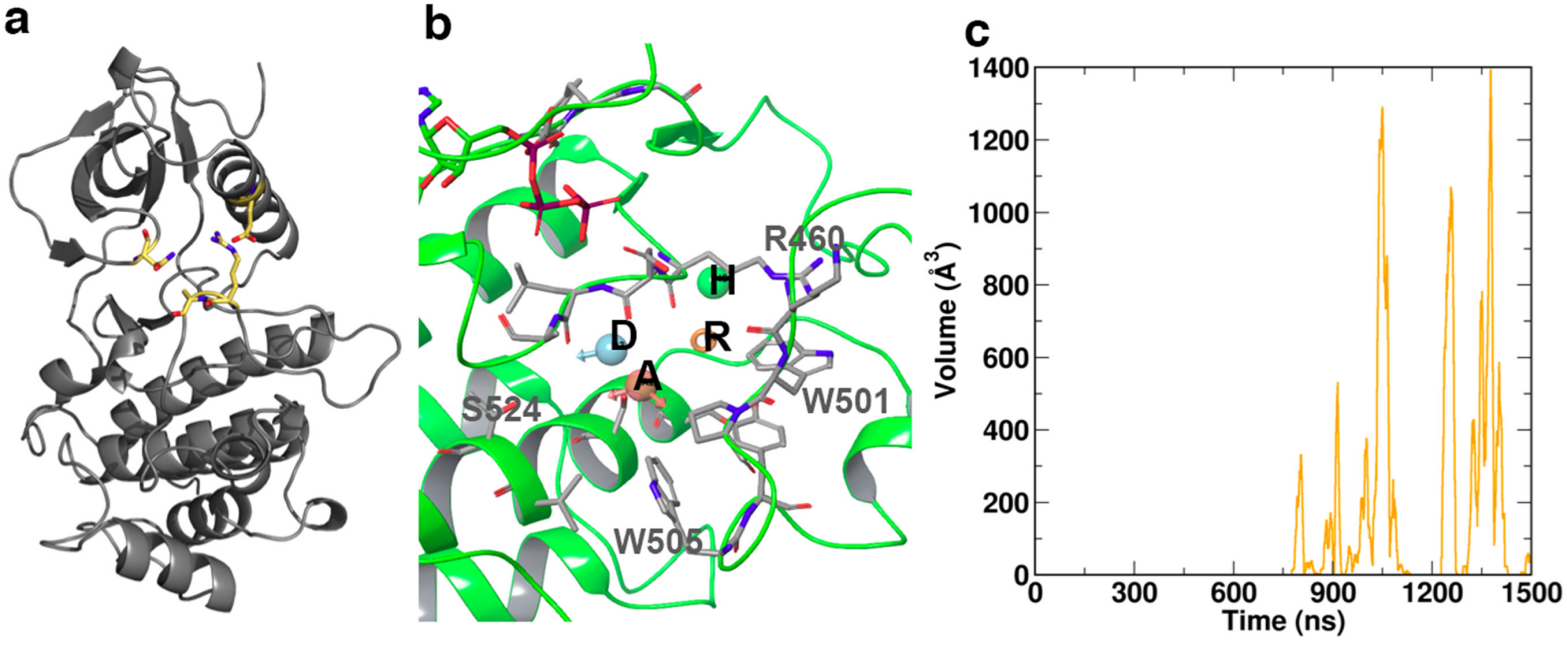
Specific structural features of the various states of ZAP70 kinase domain: (a) The salt bridge D379-R496 and the intermittent hydrogen bonding between N348-S497 (yellow) connect the C- and N-lobes and restrict access to the catalytic cleft. Additionally these bonds restrict activation loop positioning. (b) The cryptic pocket adjacent to the activation loop spontaneously opened during simulation and was unique to the non-phosphorylated state. Key residues lining the pocket are indicated in gray. Four consensus pharmacophore features were identified by fragment screening. These comprise the acceptor A, donor D, ring R, and a hydrophobic feature H, indicated inset. (c) The cryptic pocket repeatedly opened and closed over the final ~1μs of simulation time, reaching a maximum volume of ~1400 Å ^3^

Structural features specific for the staurosporine complex during our simulations comprise an additional salt bridge (D379-R496) formed across the catalytic cleft, and an adjacent intermittent hydrogen bond between the S497 hydroxyl and the N348 side chain amide group. This interaction is not observed in any X-ray structures of ZAP–70. Moreover, this behavior was not observed in any other complex. These interactions span the catalytic cleft and connect the N- and C-lobes, thereby significantly restricting substrate access. Moreover, these stabilize the position of the activation loop. These structural features are illustrated in Fig 4a. Additionally, an intra-strand interaction hydrogen bond between the side chains of R514 and Y493 could be discerned for significant parts of the trajectories even in the absence of Y493 phosphorylation.

### Non-phosphorylated ATP Complex Y^0^Y^0^

Non-phosphorylated ATP-bound ZAP–70 exhibited a similar pattern of flexibility to the staurosporine-inhibited complex. It remained stable and rigid, as illustrated by an RMSD that plateaus similarly to the STA system, and low B-factors (Fig 2). Both absolute values and variability of B-factors were elevated in the N-lobal regions around residues 500–519 and 537–569 (Fig 1, green) compared to the staurosporine complex (Fig 2c). Unlike the STA system, the non-phosphorylated structure did not present a closed loop within the DFG motif during simulation. The distance from the D479 carboxylate carbon to the backbone amide hydrogen of G481 was stable at ~5 Å during the entire course of the simulation (Fig 3a). The distance between the center of mass of the αC helix and the center of mass of the C-lobe increased from 14 Å to 14.5 Å within the first ~100 ns and subsequently remained constant for the duration of the simulation. This behavior is analogous to the behavior of the staurosporine complex (Fig 3b). The contact of F480 of the DFG motif with M390 in the αC helix was significantly less pronounced than in the STA complex or any phosphorylated state (S1 Fig). The salt bridge K369-E386 was present intermittently, around 50% of the total simulation time (Fig 3c).

**Fig 5:**
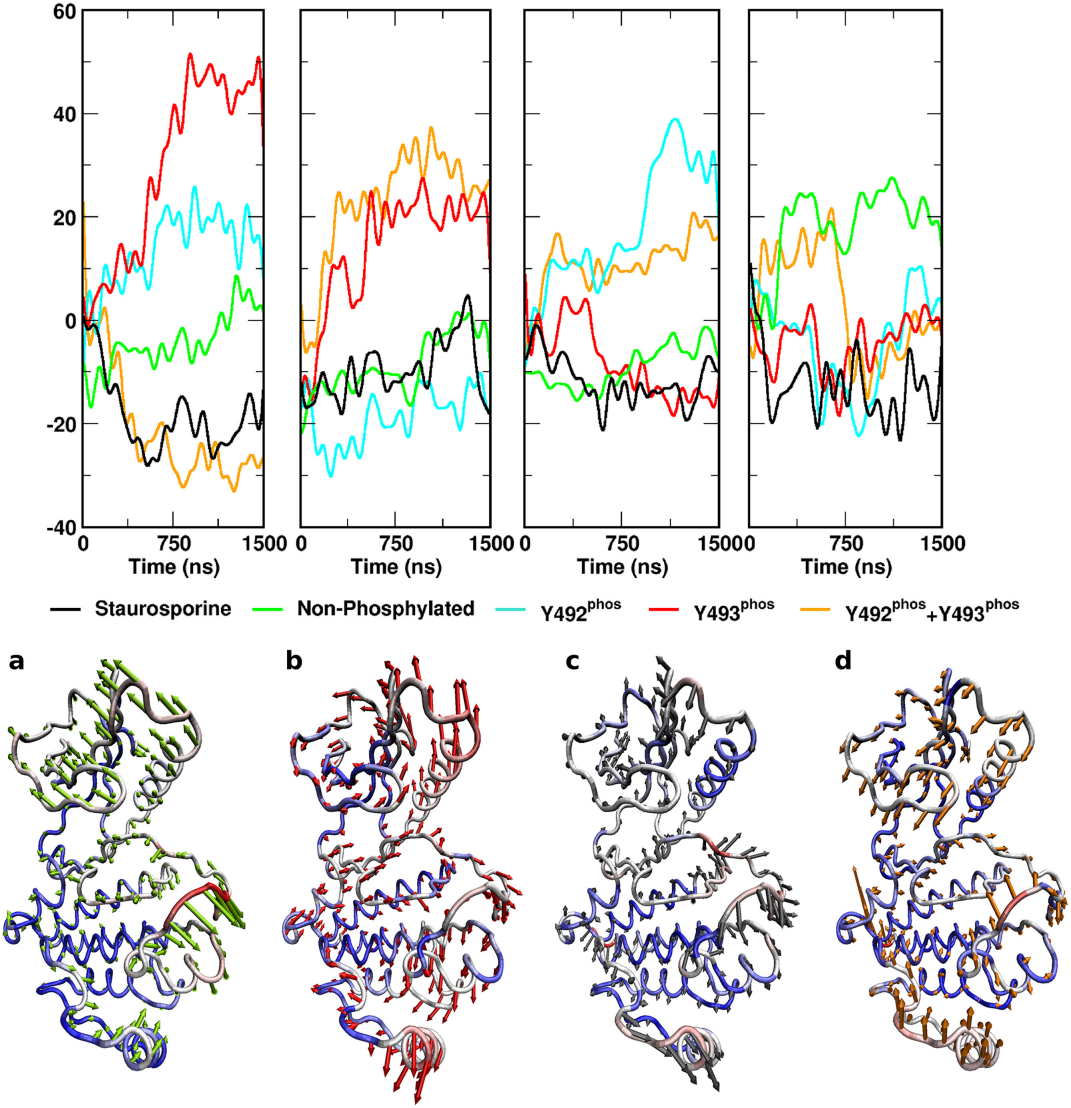
Normal mode projection amplitudes for the four lowest-frequency normal modes correlated with state of the complex. Normal mode (a) is characteristic for mono-phosphorylation as it is concurrent in the Y^P^Y^0^ and the Y^0^Y^P^ state. Normal mode (b) is associated with Y493 phosphorylation Y^0^Y^P^. Characteristic for this motion is the movement of the αC helix with concurrent extension of the activation loop. The tertiary mode (c) is characteristic for Y^P^Y^0^. It consists of an extension of the activation loop. However, no motion of the αC helix is associated with this mode. Normal mode (d) characterizes the non-phosphorylated state. Predominant motions increase compactness, close the catalytic cleft and bury the αC helix.

Fig 5 shows Normal modes and projections of the four lowest-frequency modes during the entire trajectory. Mode number 4 was specific for the non-phosphorylated complex, and is associated with a movement of the αC helix towards the remainder of the C-lobe. Concomitantly, the section of the activation loop containing phosphorylation targets Y492 and Y493 moves towards the catalytic cleft, thereby restricting access. (Fig 5d) Notably, scanning the surfaces of the KD in the non-phosphorylated complex over 1.5 μs of simulation revealed the spontaneous formation of a cryptic pocket adjacent to the activation loop (Fig 4b). This cryptic pocket repeatedly opened and closed from ~700 ns, and reached a maximum volume of ~1400 Å^3^ (Fig 4c). The protein backbone geometry of the maximum open states encountered at 1050 ns, 1257 ns and 1378 ns is identical. The formation of the pocket primarily arose from the sidechain movement of a single residue, W505, which is highly conserved across kinase domains. In the initial structure, W505 forms the core of a hydrophobic cluster; thus, its aromatic ring is wedged between P539 and P502, and is in van der Waal’s contact with V527, A463, and the alkyl groups of K504 and R465. Coupled to the motion of the activation loop, the W505 ring underwent conformational switching within the hydrophobic core (S2 and S3 Figs), with a gradual shift in its position relative to the nearby ATP site, increasing the distance of separation by up to 8 Å over the final ~700 ns (S3 Fig). In its final sidechain orientation (S3 Fig) which resulted in the formation of the fully open cryptic pocket, W505 came to rest on the surface of Y506, W523, I552, and W576, encompassing residues in or nearby to the mobile N-lobal region, whilst remaining in contact with V527 and P502.

The cryptic pocket is primarily formed by residues R460, D461, L462, A463, K500, W501, P502, W505, Y506 and S524, as outlined in Fig 4d. In order to characterize the pocket in more detail, we flexibly docked a commercial library of ~1,400 fragments spanning a molecular weight range from 150–300 into this pocket when in its most expanded states. This allowed us to identify the preference of specific regions for defined structural motifs. Following multiple rounds of fragment docking and energy minimization, we used the 100 highest scoring pooled fragments to establish a consensus pharmacophore based on commonly observed features, as depicted in Fig 4d. The five top scoring poses observed across cryptic pocket conformations are illustrated in S4 Fig. The resulting consensus pharmacophore shows an acceptor, a donor, a ring and a hydrophobic feature located within the pocket.

### Y^P^Y^0^ ATP Complex

Mutational studies previously indicated that Y492 phosphorylation might not be important in ZAP–70 biological function. [8] Simulations revealed that the tyrosine-phosphorylated variant Y^P^Y^0^ exhibited elevated flexibility across the entire protein compared to both the staurosporine and non-phosphorylated ATP complexes. The Cα-RMSD increased gradually over the first microsecond, before plateauing at ~4 Å. Consistently, the B-factor values and their variability were higher in several regions of the protein compared to the non-phosphorylated complexes (Fig 2). The DFG motif began in a conformation associated with a distance of 5 Å between the D479 carboxylate carbon and G481 backbone amide hydrogen. In contrast to the non-phosphorylated variant, this distance rapidly increased at the ~600 ns mark to 8 Å, indicating a disintegration of the DFG structure (Fig 3a), consistent with geometries observed in X-ray structures of the ANP complexes 2OZO and 4K2R. The αC helix in the Y^P^Y^0^ complex initially adopts a close conformation at a distance of 13.5 Å from the center of mass of the C-lobe. After around 500 ns, it underwent a rapid shift, increasing to a distance of 14.5 Å as observed in the inhibited and non-phosphorylated complexes (Fig 3b). The K369-E386 salt bridge was absent for the majority of the simulation (Fig 3c). Motions captured in normal modes 1 and 3 are of interest for the Y^P^Y^0^ complex. Normal mode 1 is characteristic for mono-phosphorylation. The mode consists predominantly of an outward movement of the phosphorylated region of the activation loop and a torsional motion of the C-lobe against the N-lobe. (Fig 5a) Mode 3 is characteristic of Y492 phosphorylation only. It reveals extension of the activation loop but no associated movement of the αC helix. (Fig 5c)

### Y^0^Y^P^ ATP Complex

Phosphorylation on Y493 is believed to be a decisive activating factor for the KD, in its biological context. It was also observed to show elevated flexibility over the inhibited and non-phosphorylated complexes during simulation. Flexibility was higher than Y^P^Y^0^ variant in terms of RMSD, B-factors and B-factor variability, caused primarily by increased flexibility of the activation loop (Fig 2). The structure of the equilibrated DFG motif was similar to that observed in the non-phosphorylated variant. The distance between D479 carboxylate carbon and G481 backbone amide hydrogen was constant at approximately 5 Å throughout the simulation (Fig 3a). In contrast to the previously discussed complexes, the αC helix in the Y^0^Y^P^ state adopted a distinct open conformation, indicated by an increased distance from the center of mass of the C-lobe to ~15.5 Å within the first ~100 ns (Fig 3b). Consistent with this, the salt bridge K369-E386 was absent for the duration of the simulation (Fig 3c). Strikingly, the phosphorylated Y493 residue did not interact with R514, despite the complementarity of charge. This peculiar absence of charge-charge interactions extended to the Y^P^Y^P^ state. Normal modes 1 and 2 are associated with Y^0^Y^P^. Whereas normal mode 1 is characteristic for either mono-phosphorylated state, normal mode 2 is specific for Y^0^Y^P^. Normal mode 2 consists of an extension of the activation loop and concurrent rearrangement of the αC helix. Strikingly, a general opening motion at the hinge region likely allows for easier substrate access to the catalytic cleft. (Fig 5b)

### Y^P^Y^P^ ATP Complex

The di-phosphorylated complex is characterized by the highest flexibility of all investigated states, slightly higher than Y^0^Y^P^. Cα RMSD indicates high conformational variability (Fig 2a). Analysis of the B-factors profile and associated variability revealed particularly pronounced dynamics within the activation loop and the N-lobal region comprising residues 537–569 (Fig 2b and 2c). This region is associated with the opening of the cryptic pocket observed in the non-phosphorylated Y^0^Y^0^ state. In the present Y^P^Y^P^ state, however, the segment 537–569 moves independently of the C-terminal activation loop segment, thus not forming the cryptic pocket observed in the Y^0^Y^0^ state. The DFG motif was observed to rapidly lose its structure, and spontaneously adopted an extended conformation similar to the Y^P^Y^0^ mono-phosphorylated state, with a D479 carboxylate carbon to G481 backbone amide hydrogen distance of 8 Å after approximately 50 ns. This extended conformation was present for the duration of the simulation (Fig 3a) and can also be observed in the 2OZO and 4K2R ANP-bound crystal structures. The conformation of the αC helix observed in the present state was analogous to the conformation in the Y^0^Y^P^ state, at a distance of 15.5 Å from the C-lobe center of mass (Fig 3b), whilst the salt bridge K369-E386 was not present for the duration of the trajectory. Di-phosphorylation was not represented in a singular normal mode. Generally, Y^P^Y^P^ exhibited a combination of structural and dynamic features observed individually for the Y^P^Y^0^ and Y^0^Y^P^ states.

## Discussion

### Patterns of Conformational Plasticity Associated with ZAP–70 Kinase Domain

**Fig 6:**
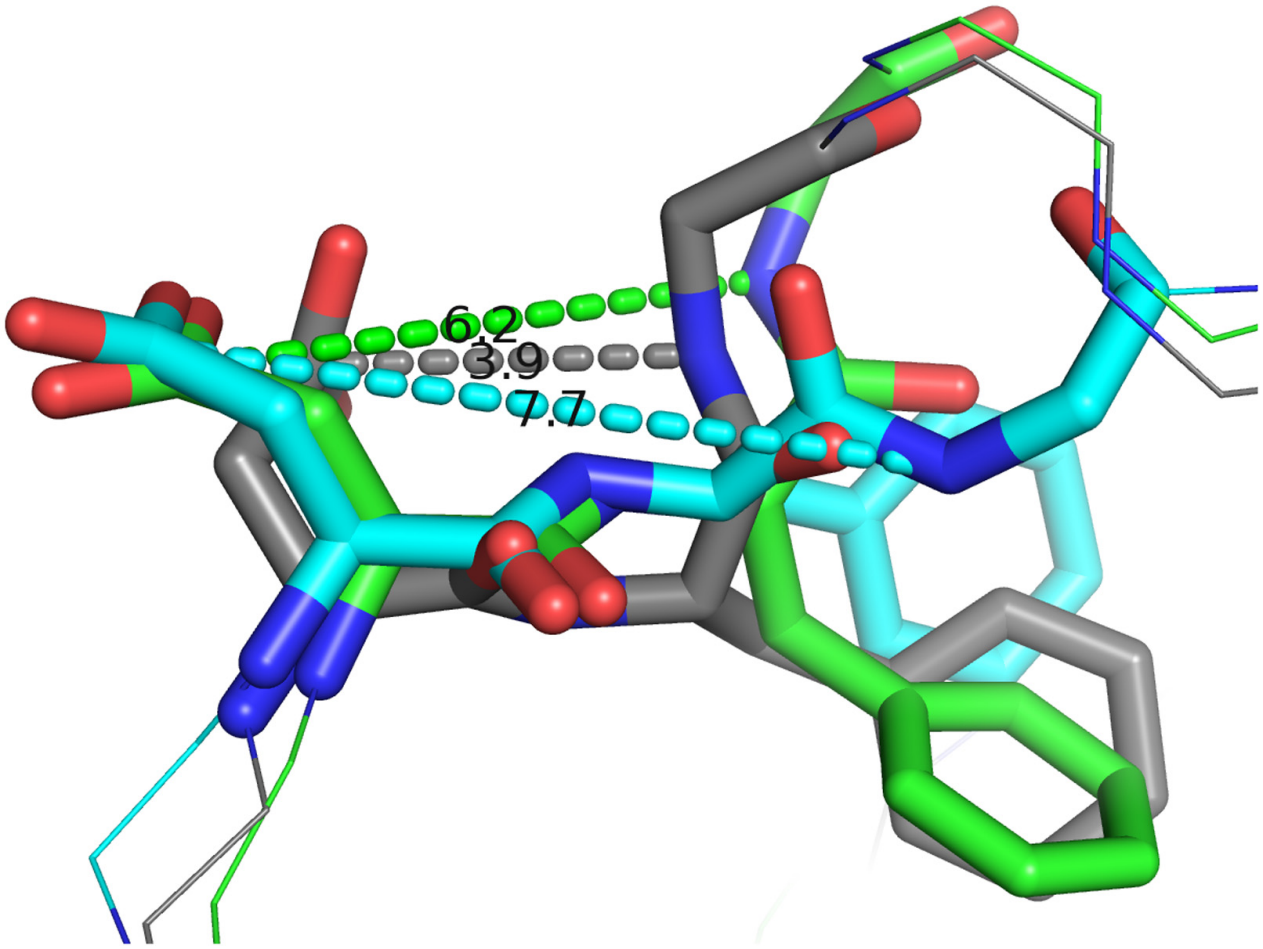
Conformations of the DFG motif: Closed (gray), semi-closed (green) and open conformation (cyan). Staurosporine causes the DFG motif in ZAP–70 to adopt the closed conformation exclusively, presumably because D479 is unable to interact with the Mg^2+^ ion coordinated by ATP. The Y^0^Y^0^ and Y^0^Y^P^ variants predominantly adopt the semi-closed form. The open extended geometry is only observed in the Y^P^Y^0^ and Y^P^Y^P^ states. Labels within the figure indicate measured distances, in Angstroms.

Common patterns observed in all simulated states were characterized by two distinct, comparatively rigid cores of the C-lobe and N-lobe, as well as flexible segments of the activation loop and the region formed by residues 537–569. Baseline flexibility in the non-phosphorylated and inhibited states was significantly lower than in either mono- or di-phosphorylated states, as indicated by the calculated B-factors. In order to differentiate between the alternative phosphorylated states, it should be noted that experimentally Y492F does not adversely affect ZAP–70 activity, whereas Y493F abolishes ZAP–70 function. [8] Therefore, we can use the effects of Y492 phosphorylation as a baseline for dynamic changes induced by monophosphorylation at the C-terminal end of the activation loop and contrast its effects with those observed in states containing phosphorylated Y493. The differences in dynamics between the two states offer some indication of the functional relevance of the observed changes across systems. Whereas phosphorylation caused a global increase in protein flexibility, specific changes were localized around the activation loop as well as the αC helix region. Strikingly, the conserved DFG motif at the N-terminal side of the activation loop adopted three distinct states across the different simulation systems, characterized by a cyclization through hydrogen bonding. By considering the distance between the carboxylate carbon of D479 and the backbone amide hydrogen of G481, we could identify a cyclic, closed state at approximately 3 Å, a semi-closed state at a distance of ~6 Å, and an open state at a distance of ~8 Å (Fig 6). We surmise that the semi-closed state of the DFG motif is relevant for the catalytic activity in ZAP–70 as it occurs in the un-phosphorylated state as well as in the Y^0^Y^P^ state. Y^P^Y^P^ and Y^P^Y^0^ bias the DFG conformation towards the open state whereas staurosporine inhibition results in stabilization of the closed state. We postulate that Y^P^Y^0^ causes a repulsive charge-charge interaction with D479, thereby promoting the open state. As staurosporine is bereft of a negative charge proximal to the DFG motif and misses an Mg^2+^ ion, D479 is free to position itself in a hydrogen bonding orientation towards the backbone N-H of G481. This behavior of the DFG motif is consistent with differing orientations of D479 observed in the staurosporine-bound X-ray structure 1U59 versus the ANP-bound complexes 2OZO and 4K2R. Generally, our observations signify that residue D479 located close to the catalytic center is highly sensitive to its local electrostatic environment. It should be noted that all simulations started from the staurosporine-bound protein conformation represented by 1U59, as it is the only structure in which all residues of the activation loop are resolved. While the choice of this starting state may introduce a bias towards active-like states in the remaining simulations, the reorientation of the DFG motif is consistent with the 2OZO and 4K2R structures. Thus we assume that the simulation times are sufficient to sample at a minimum conformational transitions between the active and intermediate states [19]. Despite the length of our trajectories, we were unable to observe DFG-in/DFG-out transitions as indicated by the interaction of F480 with M390 in the αC helix (S1 Fig).

A recurring motif in Src kinase family activation patterns is the formation of a salt bridge from the bulk of the C-lobe to the αC helix. In ZAP–70 this salt bridge may form between R369 in the C-lobe and E386 in the αC helix. This connection is associated with motions of the αC helix that have previously been identified as crucial in Src kinase family active states. Intermittent closing and opening of this salt bridge was observed throughout the course of the simulation for the non-phosphorylated, ATP-bound kinase, thus indicating a fine energetic equilibrium at this state point. Inhibition by staurosporine caused this salt bridge to adopt a permanently closed position, consistent with that observed in the 1U59 crystal structure. All phosphorylated states revealed an increased average distance between the guadinium group of R369 and the E386 carboxylate function, as well as elevated variance of this distance compared to the non-phosphorylated state. Positioning of the αC helix relative to the bulk of the protein fits with this pattern. Inhibited and non-phosphorylated states show a closer positioning to the protein center of mass of the αC helix. Phosphorylation of Y492 led to a similar position of the αC helix as the inhibited and non-phosphorylated states. Only phosphorylation of Y493 or di-phosphorylation induced repositioning of the αC helix, consistent with kinase activation. From these observations we conclude that the strength of the salt bridge R369-E386 is weakened by phosphorylation and is a necessary step for subsequent repositioning of the αC helix towards an active conformation. This repositioning is solely observed if Y493 is phosphorylated. Mono-phosphorylation of Y492 weakens the salt bridge, but does not induce a conformational shift in the αC helix.

### Mechanisms of Inhibition

Inhibition of ZAP–70 was observed to be associated with a general reduction in flexibility across the KD. Moreover, all investigated structural features comprising the DFG motif, the αC helix, the R369-E386 salt bridge, as well as patterns of normal modes and B-factors, indicate lowered dynamics for the staurosporine complex. Our analyses suggest that binding of staurosporine traps the ZAP–70 KD in a compact and rigid state. The observed rigidity would limit both substrate access and the likelihood of competitive binding by co-factor at the catalytic center.

### Cryptic Pocket

Our analyses have allowed us to identify a novel pocket adjacent to the active site and the activation loop. This pocket is neither a classical type I or type II kinase pocket and offers potential possibilities for design of new specific inhibitory ligands. Interestingly, the pocket only occurs in the non-phosphorylated state. Whereas we did not expect to find it in the very compact and rigid STA complex, it is quite surprising that the pocket was not observed in the generally more flexible phosphorylated states. Closer examination of the normal modes reveals that the phosphorylated states exhibit concerted motions along the entire activation loop, whereas these motions are much more localized to the region following the two tyrosines Y492 and Y493 in the non-phosphorylated state. We therefore postulate that the opening of this pocket is facilitated by a flap-like rearrangement of residues 495–498, associated with the “gating” of the conserved residue W505, which switches to an alternative hydrophobic environment supported by residues in or nearby to the mobile N-lobal region. Strikingly, of seven known loss-of-function mutants in the ZAP–70 kinase domain reported to lead to SCID in humans [3], five are either associated with residues that initially contact W505 or reorient and interact with it during the gating process. These include R465 (two reported missense mutants), part of the highly conserved DLAARN motif, and K538 (13 base pair deletion) in the flexible loop region, both of whose alkyl groups form van der Waal’s interactions with W505, along with A507 and S518 (missense mutants) which interact with W505 and reorient during gating and cavity formation. The remaining two, M572 (missense) and K541 (splicing error), are expected to change the local environment of W505 in its final, gated state. Finally, two hypomorphic ZAP–70 kinase mutants in mice with partial defects in TCR signaling included mutation to arginine of W504 (equivalent to W505 in humans) alone or in combination with I367F [20]. Thus we surmise that drugging this cryptic pocket could prove valuable in the study of SCID. Proceeding work will focus on targeting this pocket with small-molecules and establishing the validity of our approach by experimentally probing the chemical and structural biology of this site. We presume that increasing the size of residues lining this pocket through mutational modifications F516W, D521E or S524T could bias the system towards a stable, permanently open conformation of the cryptic pocket.

### Mechanisms of Activation

**Fig 7:**
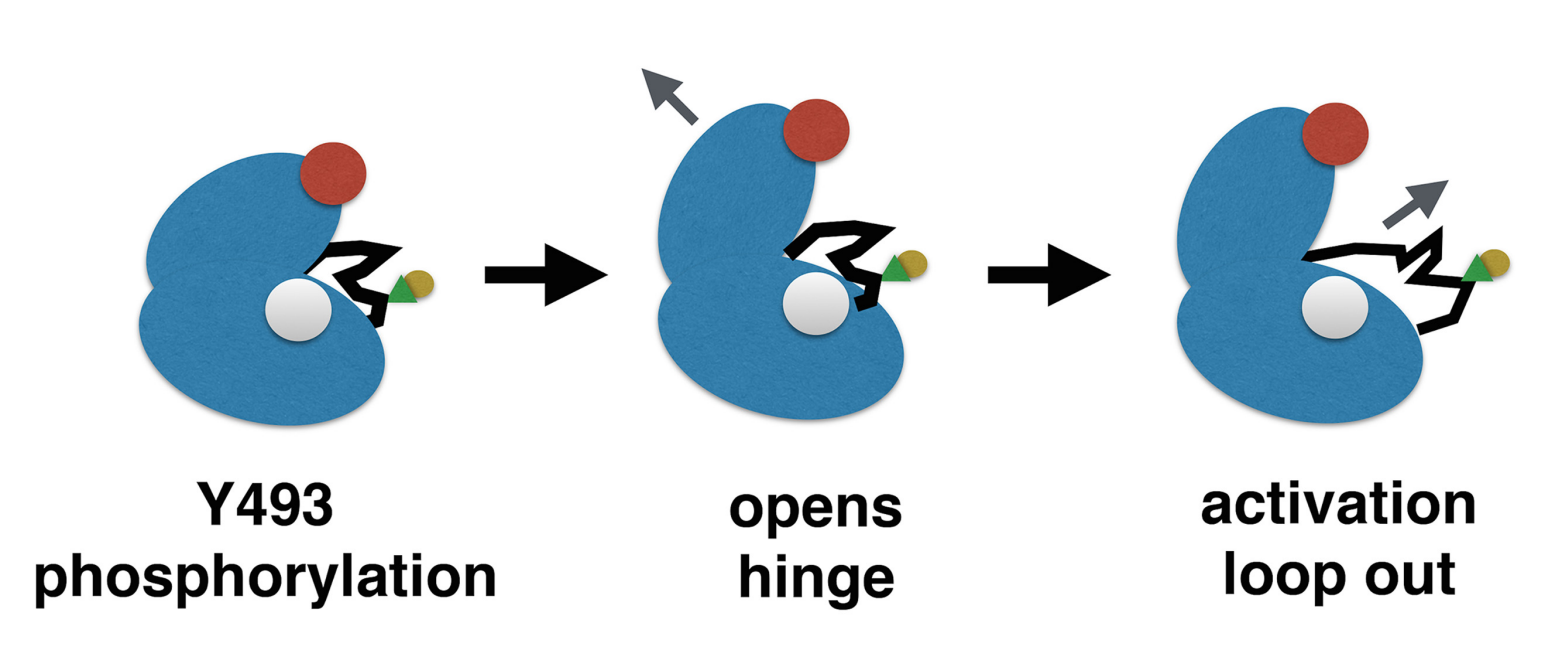
Proposed activation cascade of ZAP–70 kinase domain through the phosphorylation of Y493: upon phosphorylation of Y493 (green triangle), the salt bridge K369-E386 weakens and thus allows for movement of the αC helix (red) towards an active conformation. Concurrently, the activation loop (black) becomes more dynamic, thus allowing easier access to the active center.

Phosphorylation was observed to lead to a global increase in dynamics, irrespective of the precise phosphorylation state. Double phosphorylation at Y492 and Y493 had a significantly stronger mobilizing effect than single phosphorylation of either residue. However, structural changes were distinctly different for the individual mono-phosphorylated complexes. Mutational studies suggest that Y492 is only weakly implicated in biological activation of ZAP–70. However, Y493 phosphorylation is of crucial importance to biological function as the Y493F mutation abolishes catalytic activity. Our simulations allow us to identify changes that are specific to Y493 phosphorylation and therefore let us trace the activation cascade: Y493 phosphorylation causes the salt bridge R369-E386 connecting the C-lobe with the αC helix to weaken. In contrast to Y492 phosphorylation, Y493 phosphorylation also promotes rearrangement of the αC helix towards an active conformation already observed for members of the Src kinase family. Concurrently, flexibility of the activation loop increases significantly, thus allowing for easier access to the catalytic center. A similar increase in activation loop exposure has been observed in the activation of focal adhesion kinase (FAK). However, the activation cascade of FAK is not directly analogous to ZAP–70, as FAK has an additional FERM domain involved in forming an auto-inhibited complex. [21] Our proposed activation cascade for ZAP–70 is summarized in Fig 7. Normal mode analysis further confirms these motions as characteristic for the biologically relevant Y493 phosphorylation.

### Conclusions

In the present study, we investigated the mechanisms underlying ZAP–70 activation and inhibition. We were able to identify crucial motions associated with biological activation. We demonstrated how subtle changes in these patterns trap the enzyme in an inhibited state that superficially resembles an “active-like” structure, and derived a viable microscopic mechanism of ZAP–70 KD activation by phosphorylation. Furthermore, our simulations have allowed us to identify a cryptic pocket that is neither a classical type I nor type II binding site, and may offer promise as a new site for specific targeting by small molecule ligands.

## Methods

### System Preparation

Initial coordinates for ZAP–70 KD were obtained from the PDB [22] structure 1U59. [11] All histidine residues were set to be deprotonated and in the ε-NH tautomeric state. All other ionisable residues were set in their default, charged state. No cysteine residue is involved in disulfide bond formation. This structural template was used to construct five distinct complexes. These comprised one complex with the bound staurosporine inhibitor, and Y492 and Y493 in their non-phosphorylated states (STA). Moreover, four ATP-bound complexes were built in various phosphorylation states, namely: non-phosphorylated at Y492 and Y493 (Y^0^Y^0^), Y492 phosphorylated (Y^P^Y^0^), Y493 phosphorylated (Y^0^Y^P^)^,^ and both Y492 and Y493 phosphorylated (Y^P^Y^P^). All ATP-bound complexes were constructed by superimposing ATP and the Mg^2+^ ion from PDB 4K2R onto PDB 1U59. Parameters for staurosporine were described by CGenFF version 2b5. [23] Protein interactions were modeled using the CHARMM22/CMAP force field. [24] [25] All systems were solvated in a 0.1 M sodium chloride solution containing approximately 12,000 TIP3P [26] water molecules. Solvation resulted in rectangular box sizes of approximately 8.4 x 8.4 x 5.9 nm.

### Simulation Setup

The systems were equilibrated by performing 1000 steps of steepest descent minimization followed by a series of three 500 ps of *NpT* ensemble simulations with gradually decreasing position-restraints on the protein and ligand heavy atoms. All simulations were performed using GROMACS 4.5.5. [27] Electrostatic interactions were described using particle mesh Ewald. [28] Van-der-Waals and Ewald cut-offs were set to 1.2 nm. Bonds to hydrogen atoms were constrained with the LINCS algorithm [29] allowing an integration time step of 2 fs. Temperature was controlled for distinct coupling groups of solvent and solute using separate v-rescale thermostats [30] at 298 K, using a coupling constant τ of 1 ps. An isotropic Parrinello-Rahman barostat [31] maintained a pressure of 1 atm, using a coupling constant τ of 5 ps. Following equilibration, all systems were simulated for 1.5 microseconds in the NpT ensemble. Frames were saved every 100 ps yielding a single, continuous trajectory of 15,000 frames for each system.

### Analysis

Analysis of the trajectories was performed using *cpptraj* from the AmberTools 14 package. [32] All trajectories were aligned by their Cα atoms. Subsequently, backbone RMSD and B-factors were calculated as illustrated in Fig 2. Individual trajectories were split into 10 sequential parts of equal length, and B-factors were evaluated separately for each part. This allowed us to assess convergence, by identifying whether local flexibility and the average structure remains constant throughout the simulation or is subject to change. Regions of particular interest comprise the activation loop, the conserved DFG motif containing D479, F480 and G481, [33] and the αC helix with the associated salt bridge K369-E386. Relative positions and conformations of these regions as a function of simulation time are summarized in Fig 3. Subsequently, trajectories of all systems were concatenated and normal modes were calculated. [34] Projections were obtained and the amplitudes of these projections were correlated with the respective states. This procedure allowed us to identify crucial conformational transitions associated with each state. Normal mode components and projections for the three lowest-frequency non-zero normal modes are given in Fig 5. trj_cavity [35] was used to screen for novel transient pockets occurring during the course of the simulation.

### Fragment Screening and Pharmacophore Model

The three frames of the non-phosphorylated trajectory that show the largest volume for the cryptic pocket, at 1050 ns, 1257 ns, and 1378 ns respectively, were selected for fragment screening. These frames were imported into Schrodinger Maestro 2015–2. Subsequently, the *prepwiz* tool was used to minimize hydrogen atoms while keeping protein heavy atoms restrained. A commercial library of 1430 fragments spanning a molecular weight range from 150–300 were prepared using the *ligprep* tool to generate protomers and tautomers. A receptor grid centered on the pocket residues R460, D461, L462, A463, K500, W501, P502, W505, Y506 and S524 was generated for each of the three structures. The ligand library was docked using *GlideSP*. Ligands were allowed to be flexible during docking. Five initial states per ligand were retained, minimization was carried out and the highest-ranking pose after minimization was retained. The 100 highest scoring fragments from each structure were pooled and used as a basis to create a pharmacophore model of the cryptic pocket using *Prime*. Settings required a hypothesis of at least 50 ligands matching 3 to 5 features. We selected the pharmacophore with the highest number of fitting poses as the consensus pharmacophore.

## Supporting Information

S1 Fig Distance of the distal phenyl carbon CZ of F480 to the backbone amide nitrogen of M390 within the αC helix. Subsequent to equilibration, close contact is present for all systems with the exception of the non-phosphorylated state Y^0^Y^0^.
(TIF)

S2 Fig Minimum distance between ATP and W505 during simulation of the non-phosphorylated state Y^0^Y^0^. (TIF)

S3 Fig Cryptic pocket formation coupled to W505 gating during simulations of the non-phosphorylated state Y^0^Y^0^. Overlaid snapshots of the cryptic pocket site are shown, prior to its formation at 0 ns (grey), and along the pathway of partial to complete pocket formation at 1059 ns, 1257 ns, and 1377 ns (dark to light orange colors, respectively). Protein is shown as cartoons, and W505 is shown in wireframe format.
(TIF)

S4 Fig Top five scoring docked fragment poses in the ZAP–70 cryptic pocket. The pocket is located in the hinge region between the C-lobe and N-lobe of the protein (cartoons format), with the fragment (green) near to bound ATP (red), depicted in CPK wireframe format.
(TIF)
