# Supplementary figures and images for: The Structural Basis for Activation and Inhibition of ZAP-70 Kinase Domain
Source: PLoS Comput Biol. 2015 Oct 16;11(10):e1004560. doi: 10.1371/journal.pcbi.1004560 (PMC4608720; doi:10.1371/journal.pcbi.1004560)

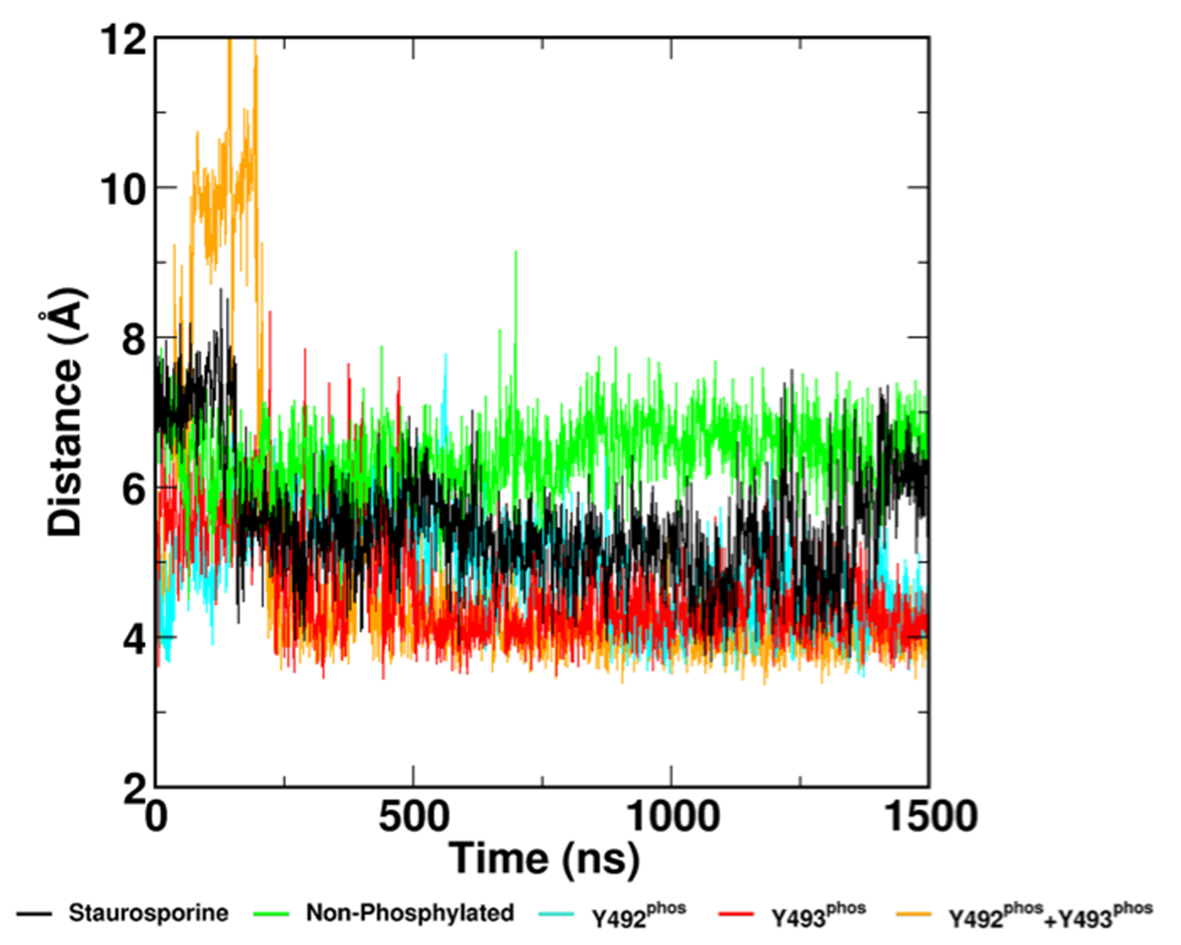

Supplement: S1 Fig — Subsequent to equilibration, close contact is present for all systems with the exception of the non-phosphorylated state Y0Y0. (TIF) [file pcbi.1004560.s001.tif]

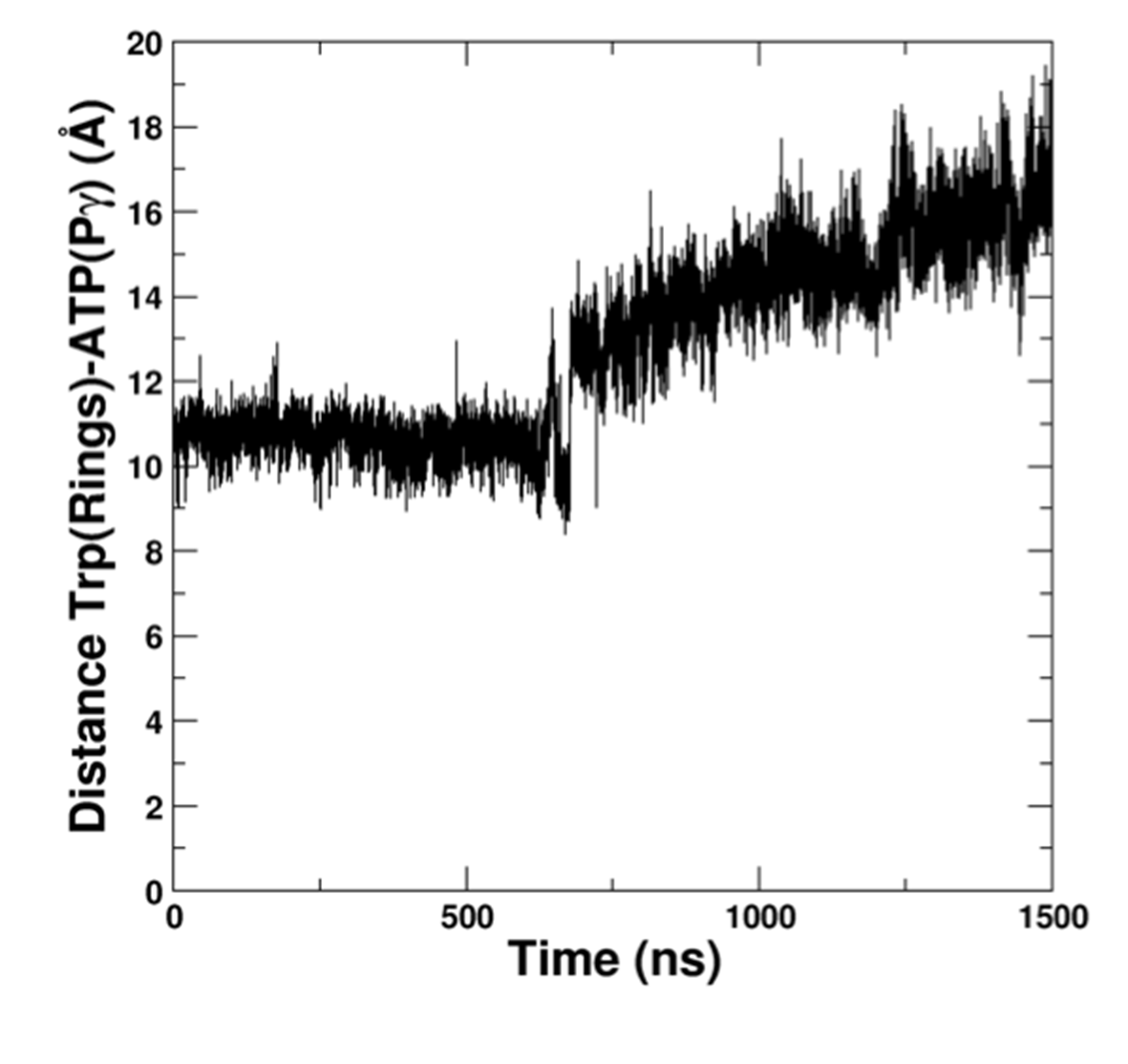

Supplement: S2 Fig — (TIF) [file pcbi.1004560.s002.tif]

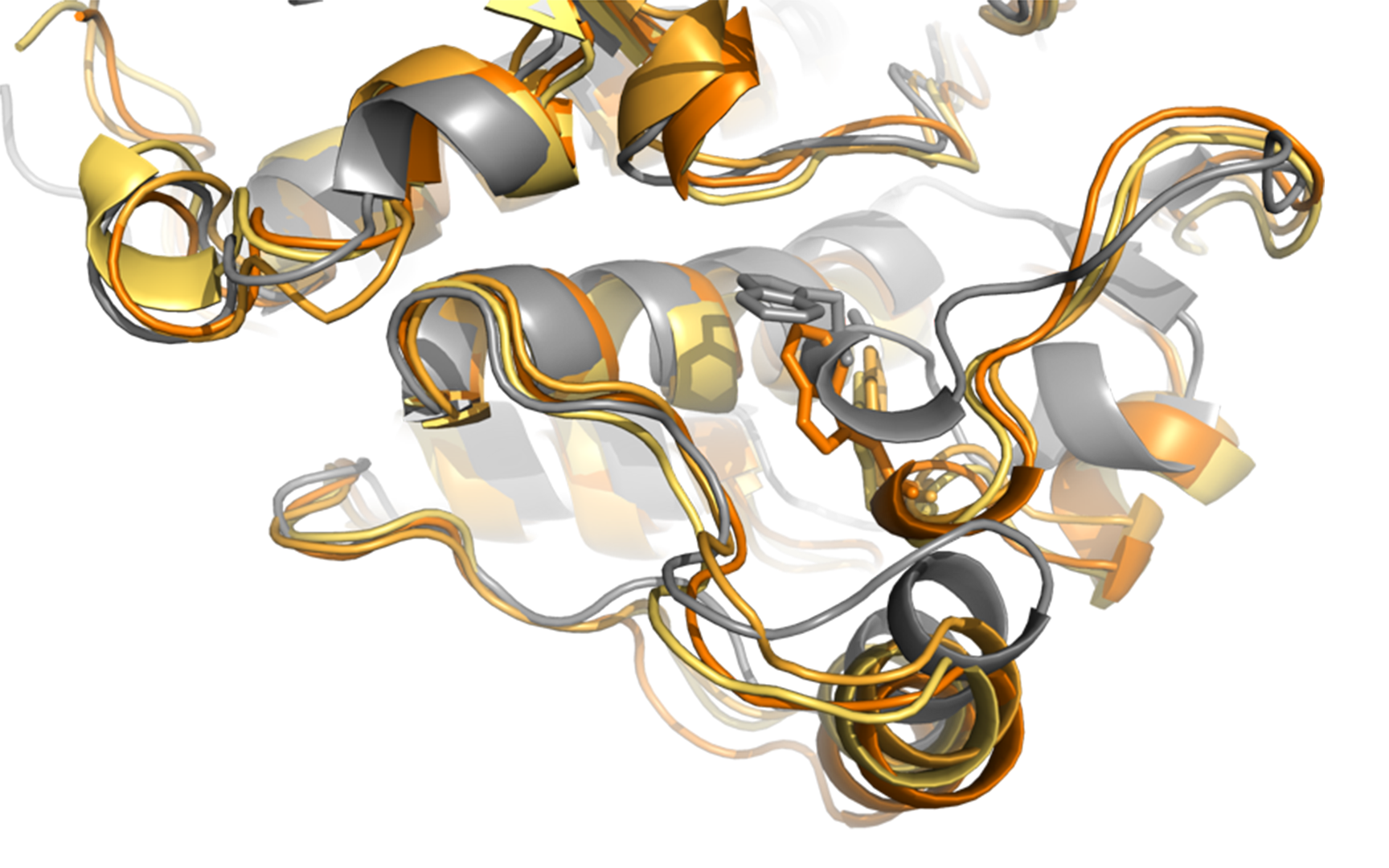

Supplement: S3 Fig — Overlaid snapshots of the cryptic pocket site are shown, prior to its formation at 0 ns (grey), and along the pathway of partial to complete pocket formation at 1059 ns, 1257 ns, and 1377 ns (dark to light orange colors, respectively). Protein is shown as cartoons, and W505 is shown in wireframe format. (TIF) [file pcbi.1004560.s003.tif]

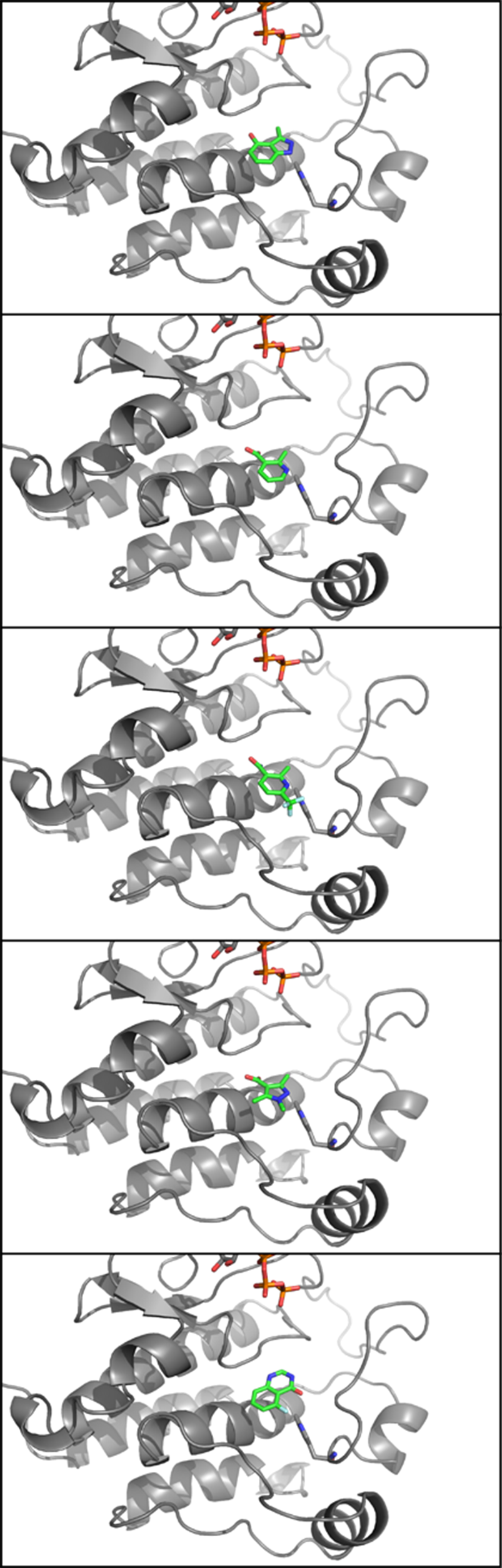

Supplement: S4 Fig — The pocket is located in the hinge region between the C-lobe and N-lobe of the protein (cartoons format), with the fragment (green) near to bound ATP (red), depicted in CPK wireframe format. (TIF) [file pcbi.1004560.s004.tif]
